# Ellagitannins of *Davidia involucrata*. I. Structure of Davicratinic Acid A and Effects of Davidia Tannins on Drug-Resistant Bacteria and Human Oral Squamous Cell Carcinomas

**DOI:** 10.3390/molecules22030470

**Published:** 2017-03-15

**Authors:** Yuuki Shimozu, Yuriko Kimura, Akari Esumi, Hiroe Aoyama, Teruo Kuroda, Hiroshi Sakagami, Tsutomu Hatano

**Affiliations:** 1 Graduate School of Medicine, Dentistry and Pharmaceutical Sciences, Okayama University, Okayama 700-8530, Japan; yshimozu@okayama-u.ac.jp (Y.S.); ph422109@s.okayama-u.ac.jp (Y.K.); pelp67gx@s.okayama-u.ac.jp (A.E.); ph20101@s.okayama-u.ac.jp (H.A.); 2 Department of Molecular Microbiology and Biotechnology, Graduate School of Biomedical and Health Sciences, Hiroshima University, Hiroshima 734-8553, Japan; tkuroda@hiroshima-u.ac.jp; 3 Division of Pharmacology, Department of Diagnostic and Therapeutic Sciences, School of Dentistry, Meikai University, Saitama 350-0283, Japan; sakagami@dent.meikai.ac.jp

**Keywords:** *Davidia involucrata*, ellagitannin, anti-bacterial effect, antitumor effect, davicratinic acid A

## Abstract

We isolated a new ellagitannin, davicratinic acid A (**5**), together with four known ellagitannins, davidiin (**1**), granatin A (**2**), pedunculagin (**3**), and 3-*O*-galloylgranatin A (**4**), from an aqueous acetone extract of dried *Davidia involucrata* leaves. The known ellagitannins were identified based on spectroscopic data. The structure of davicratinic acid A (**5**), a monomeric ellagitannin possessing a unique, skew-boat glucopyranose core, was established based on spectroscopic data. Additionally, we examined the effects of several tannins with good yields from this plant on drug-resistant bacteria and human oral squamous cell carcinomas, and found that davidiin (**1**) exhibited the most potent antibacterial and antitumor properties among the tannins examined.

## 1. Introduction

**Figure 1:**
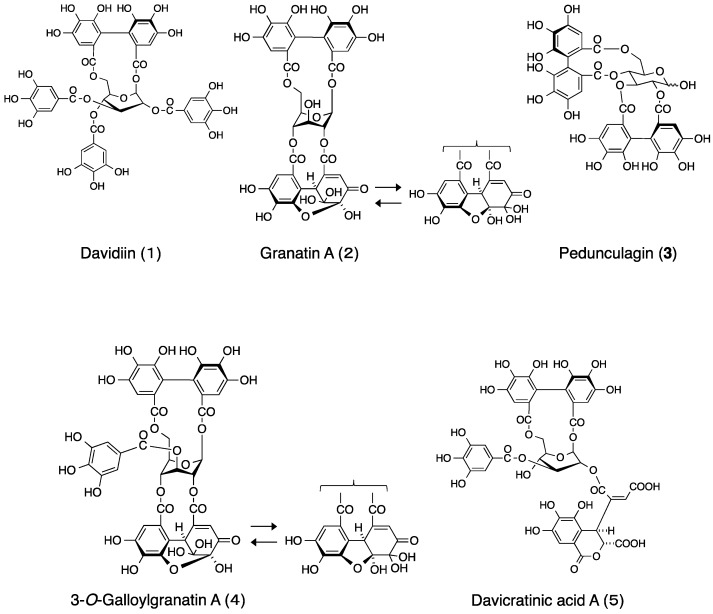
Structures of hydrolysable tannins isolated from *Davidia involucrata*.

*Davidia involucrata* Baill., known as the dove tree or handkerchief tree, is the sole species of the genus *Davidia* of the family Davidiaceae (as per the Engler system) [1]. Although it has been cultivated as an ornamental tree, its natural habitat is limited to a region in southern China. A number of constituents including phenolics [2] and triterpenes [3,4] have been identified from its branch bark. More significantly, this plant has been shown to contain ellagitannins [5]. Ellagitannins, a large group of polyphenolic compounds widely distributed in plants, are often encountered as the constituents of food, beverages, and medicinal plants [6]. Ellagitannins from various plant sources have been demonstrated to have notable antiviral, antimicrobial, and antitumor properties [7]. The isolation of davidiin (**1**) and granatin A (**2**) (Figure 1) as major tannins from *D*. *involucrata* leaves was reported in 1982 [5]. Among them, **1** is a unique ellagitannin possessing a skew-boat glucopyranose core. Recently, this compound has been identified as an inhibitor of the protein modification called SUMOylation in a post-translational process in which the inhibitor suppresses the formation of the small ubiquitin-like modifier (SUMO)-activating enzyme-SUMO-1 intermediate [8]. Despite this discovery, there are few reports of additional chemical research on its constituents, probably due to the scarcity of this plant species.

Infectious diseases caused by multidrug-resistant bacteria, including methicillin-resistant *Staphylococcus aureus* (MRSA) and vancomycin-resistant *Enterococci* (VRE), have become serious problems worldwide [9]. To date, research has been focused on developing antibacterial agents against MRSA and VRE. The synergistic effects of ellagitannins with antibiotics against drug-resistant bacteria are among the most notable antimicrobial activities of tannins [10]. Our recent studies have revealed that several phenolic compounds exhibited potent anti-VRE activity [11,12]. Accordingly, it is expected that ellagitannins from *D*. *involucrata* also have potent antibacterial effects against MRSA and VRE.

Several lines of evidence suggest that tannins have important antitumor properties, such as inhibition of tumor promotion [13,14] and inhibition of carcinogen mutagenicity [15]. Recent evidence includes the inhibitory effects of **1** on cell proliferation and tumor growth in hepatocellular carcinomas by downregulating EZH2 via a proteasome-dependent mechanism [16]. The cytotoxic effects of ellagitannin monomers and dimers from *Tamarix nilotica* against human oral squamous cell carcinomas (OSCC) [17,18,19], depending on their structures, have been shown in our previous studies.

In this study, we isolated a new ellagitannin, together with four known ones, from the leaves of *D*. *involucrata*, and determined the antibacterial effects of *Davidia* tannins on antibiotic-resistant bacteria (MRSA and VRE) and the cytotoxic effects on OSCC (Ca9-22, HSC-2, HSC-3, and HSC-4) cell lines.

## 2. Results and Discussion

### 2.1. Elucidation of the Structure of the New Ellagitannin

An aqueous acetone homogenate of the dried leaves of *D*. *involucrata* was extracted successively with chloroform, ethyl acetate, and *n*-BuOH. In addition to the four known tannins obtained from the *n*-BuOH extract, davicratinic acid A (**5**) was isolated as a new ellagitannin from the H_2_O extract. The known tannins were identified as davidiin (**1**) [7,20], granatin A (**2**) [7], pedunculagin (**3**) [21], and 3-*O*-galloylgranatin A (helioscopinin A) (**4**) [22] (Figure 1) based on the ^1^H-NMR and ESI-MS data.

**Figure 2:**
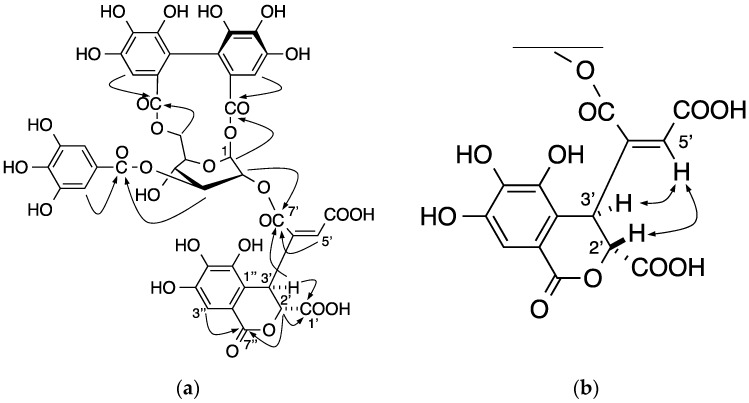
Key HMBC (**a**) and ROESY (**b**) correlations of davicratinic acid A (**5**).

**Figure 3:**
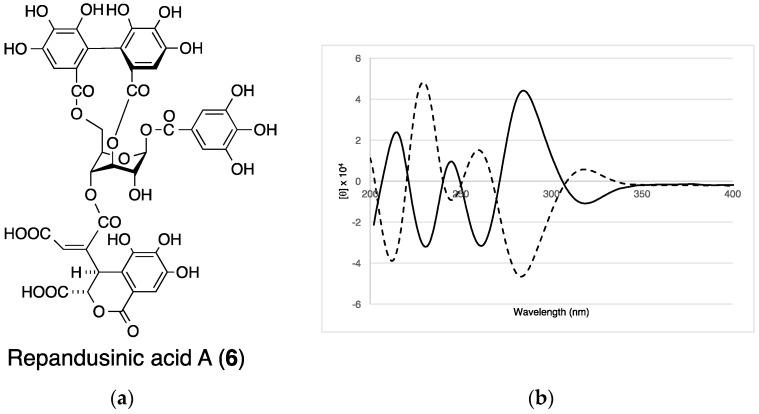
Determination of the stereochemistry of davicratinic acid A (**5**). (**a**) Structure of repandusinic acid A (**6**); (**b**) Comparison of the ECD spectra between **5** (solid line) and **6** (dashed line).

Davicratinic acid A (**5**) was obtained as a pale yellow amorphous powder. High-resolution electrospray ionization mass spectrometry (HR-ESI-MS) in the positive ion mode showed the [M + H]^+^ ion peak corresponding to the molecular formula C_41_H_31_O_28_. The ^1^H-NMR spectrum of **5** showed that the signals were due to a galloyl group (δ 7.03, 2H, s) and a 4,4′,5,5′,6,6′-hexahydroxydiphenoyl (HHDP) group (δ 6.74 and 6.89, each 1H, s). In addition, the spectrum exhibited sugar signals at δ 3.93 (1H, dd, *J* = 4.8, 7.8 Hz), 4.04 (1H, dd, *J* = 4.8, 11.4 Hz), 4.26 (1H, dt, *J* = 4.8, 11.4 Hz), 4.60 (1H, t, *J* = 11.4 Hz), 5.13 (1H, dd, *J* = 3.6, 7.8 Hz), 5.37 (1H, t, *J* = 7.8 Hz), and 5.88 (1H, d, *J* = 3.6 Hz), which were assigned to the glucose H-4, H-6a, H-5, H-6b, H-2, H-3, and H-1 signals based on ^1^H-^1^H-COSY. The appearance of the H-4 signal in the higher field indicated that the C-4 hydroxyl group was unacylated. According to the coupling constants of these glucose proton signals, the glucopyranose ring in **5** was assigned a skew-boat–type conformation. The HMBC spectrum revealed that glucose *O*-3 was acylated by the galloyl group and the *O*-1 and *O*-6 positions of glucose were bridged via the HHDP group (Figure 2a). The HMBC spectrum of **5** also showed couplings of the singlet protons at δ 6.83 (H-5′) and 5.49 (H-3′) with a carboxyl carbon signal at δ 165.5 (C-7′) of the remaining acyl group. Because this carboxyl carbon signal was further correlated with the glucose H-2 signal, the acyl group containing these protons and carbons is connected to glucose *O*-2. The HMBC spectrum also showed correlations with the methine proton signals at δ 5.15 (H-2′) and 5.49 (H-3′) with a carboxyl carbon signal at δ 171.4 (C-1′), and those of two methine proton signals (δ 5.15 (H-2′), 7.10 (H-3″)) with a carboxyl carbon signal (δ 166.8 (C-7″)). These spectral observations led us to formulate a dehydrochebuloyl group for the acyl group at glucose *O*-2. H-5′ showed an ROE correlation between H-2′ and H-3′, indicating that the geometrical isomerism of the double bond was Z (Figure 2b). To determine the stereochemistry of **5**, the ECD spectra of **5** and repandusinic acid A (**6**) (Figure 3a) [23], which also possesses the galloyl, HHDP, and dehydrochebuloyl groups, were compared. As shown in Figure 3b, the spectrum of **5** displayed the ECD pattern with the opposite signs of that of **6**. Therefore, the stereochemistry of the HHDP group in **5** is *S*, whereas both C-2′ and C-3′ of the dehydrochebuloyl group are *R*. Based on the above data, **5** was identified as 1,6-*O*-(*S*)-HHDP-2-*O*-(2′*R*,3′*R*)-(4′-dehydrochebuloyl)-3-*O*-galloyl-β-d-glucose (The data are also given in Figures S1–S7 in the supplementary material).

### 2.2. Biological Effects of Davidia Tannins

#### 2.2.1. Antibacterial Effects

To clarify the biological properties of *Davidia* tannins, we examined the major tannin **1** and related tannins **2** and **4**, obtained in high yield from *D*. *involucrata*, for antibacterial effects on MRSA (*S*. *aureus* OM481 and OM584) and VRE (*E*. *faecium* FN-1 and *E*. *faecalis* NCTC12201) by the liquid dilution method [24]. The results are summarized in Table 1, and **1** and **4** exhibited antibacterial effects on both MRSA (64 µg/mL) and VRE (16–64 µg/mL). In particular, compound **1** showed the most potent effect on *E*. *faecalis* NCTC12201. The galloyl group is related to antibacterial properties; **1**, which possesses three galloyl groups, exhibited the most potent activity (MIC 64 µg/mL for MRSA, 16 µg/mL for VRE). Conversely, compound **2**, which is composed of HHDP and dehydrohexahydroxydiphenoyl (DHHDP) groups but no galloyl groups, was ineffective. It has been reported that the extract containing ellagitannins of *Acalypha wilkesiana* var. *macafeana* exerts antibacterial effects by causing cell wall damage which eventually results in cell lysis [25]. Besides, the significance of the galloyl group in polyphenols on the instability of cell membrane was also reported [26]. Hence, there is a possibility that **1** and **4** exhibited antibacterial activities by the disruption of the cell membrane. Further studies using other bacterial strains/species and those clarifying the mechanisms for the antibacterial effects are needed.

#### 2.2.2. Cytotoxic Effects

Among various types of tannins, dimeric ellagitannins specifically displayed host-mediated antitumor activity against sarcoma-180 in mice [27]. Recently, in vitro studies using tumor cell lines have shown that monomeric, dimeric, and oligomeric ellagitannins show potent cytotoxicity against carcinoma cell lines and lower cytotoxicity to normal cells [17,18,19,28]. It has also been reported that **1**, a monomeric ellagitannin, inhibits cell proliferation and tumor growth in hepatocellular lines [16]. Thus, we also examined **1** and **4**, which showed antibacterial activities, for their cytotoxic activities on human OSCC (Ca9-22, HSC-2, HSC-3, and HSC-4) compared with their activities on human oral normal cells (HGF, HPC, and HPLF).

As shown in Table 2, both **1** and **4** exhibited moderate cytotoxicity toward OSCC. In addition, **1** showed a higher tumor-specificity (TS) index than **4**. Although the effect of **1** (TS = 2.2) was lower than several oligomeric tannins (TS = 2.1–7.1) [17,18,19], its activity was higher than that of other types of natural polyphenols (flavones, flavonols, and isoprenylated flavonoids) (TS = 1.2–2.3) and terpenes (triterpene aglycones and triterpene glycosides) (TS = 1.2–1.6) [29]. It should be noted that it is comparable with that of resveratrol, which is a natural potent antitumor compound (TS = 2.9). It seems that a larger number of phenolic hydroxyl groups on galloyl and related acyl groups in hydrolyzable tannins increase the tumor specificity. However, our previous study suggested that pentagalloylglucose is non-cytotoxic practically [28]. Therefore, these results suggest that the tumor specificity of tannins depends largely on the structure, and especially the skew-boat glucopyranose core affecting the orientations of the acyl groups may play an important role in the cytotoxicity.

## 3. Materials and Methods

### 3.1. General Experimental Procedures

One-dimensional (1D) (^1^H and ^13^C) and two-dimensional (2D) (^1^H-^1^H COSY, ROESY, HSQC, and HMBC) NMR spectra were recorded on a Varian NMR system 600 (600 MHz for ^1^H and 151 MHz for ^13^C, Agilent Technologies, Santa Clara, CA, USA). Chemical shifts are reported in δ (ppm) values relative to that of the solvent signal (acetone-*d*_6_ (δ_H_ 2.04; δ_C_ 29.8)) on the tetramethylsilane scale. Optical rotations were recorded on a JASCO DIP-1000 digital polarimeter. UV spectra were recorded using a Shimadzu UV-1800 spectrometer (Shimadzu, Kyoto, Japan). The electronic circular dichroism (ECD) spectra were measured using a JASCO J-720W spectrometer (JASCO, Tokyo, Japan). High-resolution mass spectra were obtained using the MS system QTOF G6520 (Agilent Technologies) equipped with the HPLC-Chip Cube system G4240 (Agilent Technologies). The solvent used was H_2_O–CH_3_CN (1:1, *v/v*) containing 0.1% HCOOH.

### 3.2. Materials

Dried *D*. *involucrata* leaves were kindly provided by Dr. T. Yamaura, Yamashina Botanical Research Institute (Nippon Shinyaku Co. Ltd., Kyoto, Japan). Repandusinic acid A (**6**) was prepared from geraniin [30]. Toyopearl HW-40 (coarse and fine grades; Tosoh Corporation, Tokyo, Japan), YMC-gel ODS-A (S, 75 µm; YMC), Sepabeads SP700, Dia-ion HP-20, and MCI-gel CHP-20P (Mitsubishi Chemical, Tokyo, Japan) were used for column chromatography. Dulbecco’s modified Eagle’s medium (DMEM) was obtained from GIBCO (BRL, Grand Island, NY, USA). Fetal bovine serum (FBS) and 3-(4,5-dimethylthiazol-2-yl)-2,5-diphenyltetrazolium bromide (MTT), were purchased from Sigma-Aldrich Inc. (St. Louis, MO, USA). Human normal oral cells [(gingival fibroblast (HGF), periodontal ligament fibroblast (HPLF), and pulp cells (HPC)) were established from the first premolar tooth extracted from the lower jaw of a 12-year-old girl [31]. Human oral squamous cell carcinoma (OSCC) cell lines (Ca9-22, HSC-2, HSC-3, and HSC-4) were purchased from Riken Cell Bank (Tsukuba, Japan).

### 3.3. Isolation of Davicratinic Acid A (***5***)

Dried *D*. *involucrata* leaves (230 g) were homogenized in 70% aq. acetone at room temperature to yield aq. acetone extract (68.0 g). A part (20.0 g) of the obtained extract was re-dissolved in H_2_O and the solution was extracted with chloroform, ethyl acetate, and *n*-BuOH saturated with water, successively. The *n*-BuOH extract (3.6 g) was subjected to column chromatography over Sepabeads SP700 with increasing concentrations of MeOH in H_2_O. The eluate (184.1 mg) with 30% aq. MeOH was subjected to column chromatography over YMC-gel ODS-A with aq. MeOH to yield granatin A (**2**) (31.4 mg). The 40% aq. MeOH eluate from the Sepabeads column was re-chromatographed over YMC-gel ODS-A with aq. MeOH followed by preparative HPLC to afford pedunculagin (**3**) (1.0 mg). The eluate (664.0 mg) from 60% aq. MeOH from the Sepabeads column was fractionated and purified by re-chromatography over Toyopearl HW-40C and YMC-gel ODS-A with aq. MeOH to give davidiin (**1**) (47.0 mg) and 3-*O*-galloylgranatin A (**4**) (12.9 mg). The H_2_O extract (8.1 g) obtained after the *n*-BuOH extraction was dissolved in H_2_O and the aq. solution was acidified to <pH 3 with 1 M HCl, and was subjected to column chromatography over Diaion HP-20 with aq. MeOH. The obtained 20% MeOH eluate from the Diaion column (718.5 mg) was fractionated and purified by chromatography over YMC-gel ODS-A (with aq. MeOH), Toyopearl HW-40F (with 70% EtOH), and MCI-gel CHP-20P (with aq. MeOH), followed by preparative HPLC, to yield davicratinic acid A (**5**) (8.2 mg).

*Davicratinic acid A* (**5**): Pale-yellow amorphous powder; [α]${}_{D}^{19}$ −4.9 (*c* = 1.0, MeOH); UV λ_max_ (MeOH) nm (ε): 219 (0.621), 275 (0.252); ECD (MeOH) [θ] (nm) −2.7 × 10^4^ (200), +2.8 × 10^4^ (213), −2.7 × 10^4^ (231), +2.3 × 10^4^ (244), −2.5 × 10^4^ (261), +4.4 × 10^4^ (284), −1.0 × 10^4^ (319); ^1^H-NMR (acetone-*d*_6_/D_2_O = 9/1) δ: 3.93 (1H, dd, *J* = 4.8, 7.8 Hz, H-4), 4.04 (1H, dd, *J* = 4.8, 11.4 Hz, H-6a), 4.26 (1H, dt, *J* = 4.8, 11.4 Hz, H-5), 4.60 (1H, t, *J* = 11.4 Hz, H-6b), 5.13 (1H, dd, *J* = 3.6, 7.8 Hz, H-2), 5.15 (1H, br s, H-2**′**), 5.37 (1H, t, *J* = 7.8 Hz, H-3), 5.49 (1H, br s, H-3**′**), 5.88 (1H, d, *J* = 3.6 Hz, H-1), 6.74 (1H, s, HHDP-3**′**), 6.83 (1H, s, H-5**′**), 6.89 (1H, s, HHDP-3), 7.03 (2H, s, galloyl-H), 7.10 (1H, s, H-3**″**). ^13^C-NMR (acetone-*d*_6_/D_2_O = 9/1) δ: 34.7 (C-3**′**), 65.1 (C-6), 68.9 (C-4), 72.0 (C-2), 72.2 (C-3), 78.2 (C-5), 78.8 (C-2**′**), 95.2 (C-1), 108.8 (HHDP-3**′**), 108.9 (C-3**″**), 109.3 (HHDP-3), 110.0 (2C, C-2, 6), 115.0 (C-1**″**), 115.9 (HHDP-1), 116.1 (HHDP-1**′**), 116.9 (C-2**″**), 120.2 (galloyl-1), 124.8 (HHDP-2), 125.4 (HHDP-2**′**), 136.4 (HHDP-5), 136.6 (HHDP-5**′**), 137.3 (C-4**′**), 137.5 (C-5**′**), 139.3 (galloyl-4), 139.5 (C-5**″**), 144.0 (C-6**″**), 144.3 (2C, HHDP-4, 4**′**), 144.6 (HHDP-6), 144.9 (HHDP-6**′**), 145.3 (C-4**″**), 145.7 (2C, galloyl-3, 5), 165.5 (C-7**′**), 166.8 (C-7**″**), 166.9 (2C, C-1, 3), 168.8 (2C, C-6, 6**′**), 171.4 (C-1**′**). HR-ESI-MS *m*/*z*: 971.0952 [M + H]^+^ (calculated for C_41_H_31_O_28_ + H, 971.0996).

### 3.4. Antibacterial Assay

Two strains of MRSA, OM481 and OM584, clinical isolates from Okayama University Hospital that were stored in the Department of Microbiology Laboratory, were used in this study. *Enterococcus faecium* FN-1 and *E*. *faecalis* NCTC 12201 used in this study were provided by Prof. Y. Ike, Gunma University. The bacterial cells were pre-cultured in Mueller-Hinton broth at 37 °C under aerobic conditions. They were incubated in the presence of compounds with final concentrations 16, 32, 64, and 128 µg/mL, obtained by two-fold serial dilution at 37 °C without shaking, in the same broth, for 24 h on 96-well plates, as shown in a previous study [24,32]. The inoculates were adjusted to yield final cell density of about 10^4^ CFU. The lowest concentration at which visible growth was completely inhibited was regarded as the minimum inhibitory concentration (MIC). The same experiment was repeated at least three times and the values reproduced were given in Table 1. Dimethyl sulfoxide (DMSO) was used for dissolving compounds, and the final concentrations were set at <5%, where DMSO had no effect on bacterial growth. The positive control, linezolid, was used after dissolution in water.

### 3.5. Assay for Cytotoxic Activities

Human normal oral cells (HGF, HPLF, HSC) and OSCC (Ca9-22, HSC-2, HSC-3, HSC-4) were cultured at 37 °C in DMEM supplemented with 10% heat-inactivated FBS, 100 units/mL of penicillin G, and 100 µg/mL of streptomycin sulfate under a humidified 5% CO_2_ atmosphere. Cells were then harvested by treatment with 0.25% trypsin-0.025% EDTA-2Na in PBS (−) and either subcultured or used for experiments. The cells were inoculated at 2.5 × 10^3^ cells/0.1 mL in 96-well microplates. After 48 h, the medium was removed and replaced with 0.1 mL of fresh medium containing various concentrations of a test compound with three replicate wells. Each test compound was dissolved in DMSO at a concentration of 40 mM. The first well containing 400 µM of the test compound was sequentially diluted two-fold. Control cells were treated with the same amounts of DMSO, and the cell damage induced by vehicle was corrected. The cells were incubated for an additional 48 h, and the relative viable cell number was determined by the MTT method [33,34]. In brief, the treated cells were washed with phosphate-buffered saline and were incubated for another 3 h in fresh culture medium containing 0.2 mg/mL MTT. Cells were then lysed with 0.1 mL of DMSO and the absorbance of the cell lysate at 540 nm was determined using a microplate reader (Bichromatic Labsystem, Helsinki, Finland). Absorbance of the control cells ranged from 0.40 to 0.90. The 50% cytotoxic concentration (CC_50_) was determined from the dose-response curve and the mean value of CC_50_ for each cell type was calculated from three independent experiments. The antitumor potential was evaluated by the tumor-specificity index (TS). TS value was calculated by the following equation: TS = mean CC_50_ against normal cells/mean CC_50_ against tumor cells that is [CC_50_ (HGF) + CC_50_ (HPLF) + CC_50_ (HPC)]/[CC_50_ (Ca9-22) + CC_50_ (HSC-2) + CC_50_ (HSC-3) + CC_50_ (HSC-4)] × 4/3. We have previously confirmed that the TS value determined by this method reflects the antitumor potential of test samples, although these normal oral cells and OSCC cell lines are classified as mesenchymal or epithelial cells [34].

## 4. Conclusions

The findings obtained in the present study indicate that *D*. *involucrata* is an important tannin-rich resource. In particular, ellagitannin **1**, which possesses the unique skew-boat–type glucopyranose core, is abundant in this plant. In this study, we investigated new ellagitannins of this plant, and characterized the new tannin **5**, which also has a skew-boat glucopyranose core. We consider that **5** is metabolized from **4** via oxidation of the DHHDP moiety [35], and have shown that **1** is a prominent antibacterial compound. The anti-MRSA activity of ellagitannins has been previously reported [10]; to the best of our knowledge, the anti-VRE activity of tannins has not been demonstrated before this study. Although the mechanisms for the cytotoxicity remain unclear, we expect that **1** is a possible candidate for a lead compound for antitumor agents. Further studies will be needed to isolate other ellagitannins related to **1** from *D*. *involucrata*, and will be important in understanding the tannin metabolism in plants; examination of the structure-activity relationship of these tannins on antibacterial and antitumor effects will also be important.

## Figures and Tables

**Table 1:** Antibacterial effects of *Davidia* tannins. ^a^

Compounds	MIC (µg/mL)			
	MRSA		VRE	
	*S. aureus*		*E. faecium*	*E. faecalis*
	OM481	OM584	FN-1	NCTC12201
**1**	64	64	>128	16
**2**	128	128	>128	128
**4**	64	64	>128	64
Linezolid	2	1	2	2

^a^ The acronyms used are: MIC, minimum inhibitory concentration; MRSA, methicillin-resistant *Staphylococcus aureus*; VRE, vancomycin-resistant *Enterococci*

**Table 2:** Cytotoxicity of *Davidia* tannins against human normal and tumor cells. ^a^

Compounds	CC_50_ ± SD (µM) ^b,c^							
	Human Tumor Cells				Human Normal Oral Cells			
	Ca9-22	HSC-2	HSC-3	HSC-4	HGF	HPLF	HPC	TS ^d^
**1**	140 ± 8.7	112.3 ± 11.2	99.6 ± 8.6	152.7 ± 13.6	254.7 ± 16.3	309.0 ± 12.8	256 ± 28.8	2.2
**4**	97.3 ± 9.0	111.0 ± 3.5	114.7 ±14.5	95.3 ± 22.3	128.7 ± 3.8	155.0 ± 11.4	131.0 ± 1.7	1.3
Resveratrol	106 ± 6.0	71.0 ± 0.6	67.7 ±1.3	46.2 ± 6.9	215.0 ± 29.7	198.3 ± 22.3	214.0 ± 12.1	2.9

^a^ See also Table S1 in the supplementary material; ^b^ CC_50_, 50% cytotoxic concentration; ^c^ Each value represents the mean from three independent experiments; ^d^ TS, tumor-specificity index.

## Supplementary Materials

Supplementary materials are available online.
