# Supplementary material for: Ellagitannins of Davidia involucrata. I. Structure of Davicratinic Acid A and Effects of Davidia Tannins on Drug-Resistant Bacteria and Human Oral Squamous Cell Carcinomas
Source: Molecules. 2017 Mar 15;22(3):470. doi: 10.3390/molecules22030470 (PMC6155176; doi:10.3390/molecules22030470)
Supplement: Supplementary file 1 [file molecules-22-00470-s001.pdf]

Supporting information for

Ellagitannins of *Davidia involucrata*. I. Structure of Davicratinic Acid A, and Effects of Davidia Tannins on Drug-resistant Bacteria and Human Oral Squamous Cell Carcinoma.

Yuuki Shimozu[1], Yuriko Kimura[1], Akari Esumi[1], Hiroe Aoyama[1], Teruo Kuroda[2], Hiroshi Sakagami[3], Tsutomu Hatano[1]

1 Graduate School of Medicine, Dentistry and Pharmaceutical Sciences, Okayama University, Okayama 700-8530, Japan
2 Department of Molecular Microbiology and Biotechnology, Graduate School of Biomedical and Health Sciences, Hiroshima University, Hiroshima 734-8553, Japan
3 Division of Pharmacology, Department of Diagnostic and Therapeutic Sciences, School of Dentistry, Meikai University, Saitama 350-0283, Japan

Corresponding author: hatano@pharm.okayama-u.ac.jp

List of contents

^{1}H NMR spectrum of davicratinic acid A (**5**) in acetone-d_6/D_2O=9/1. ……….…..... Figure S1

^{13}C NMR spectrum of davicratinic acid A (**5**) in acetone-d_6/D_2O=9/1. …………... Figure S2

^{1}H-^{1}H COSY spectrum of davicratinic acid A (**5**) in acetone-d_6/D_2O=9/1. ……….. Figure S3

HSQC spectrum of davicratinic acid A (**5**) in acetone-d_6/D_2O=9/1. ……………..….. Figure S4

HMBC spectrum of davicratinic acid A (**5**) in acetone-d_6/D_2O=9/1. ……………….. Figure S5

ROESY spectrum of davicratinic acid A (**5**) in acetone-d_6/D_2O=9/1. ……………… Figure S6

(+)-HR-ESI-MS spectrum of davicratinic acid A (**5**). ....…… Figure S7

Cytotoxicity of the *Davidia* tannins...................................……............................. Table S1

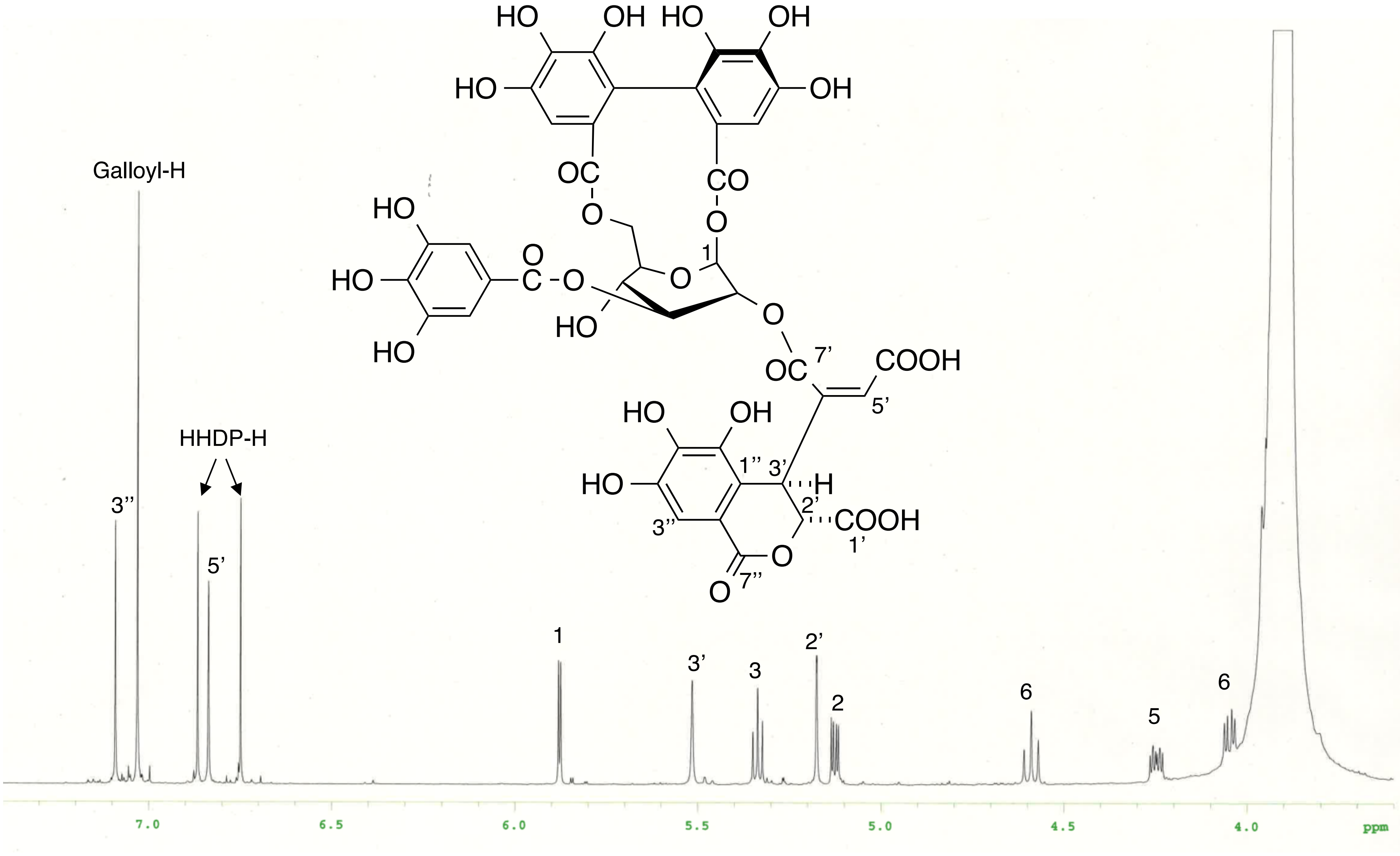

Figure S1. ^{1}H NMR spectrum of davicratinic acid A (**5**) in acetone-d_6/D_2O=9/1.

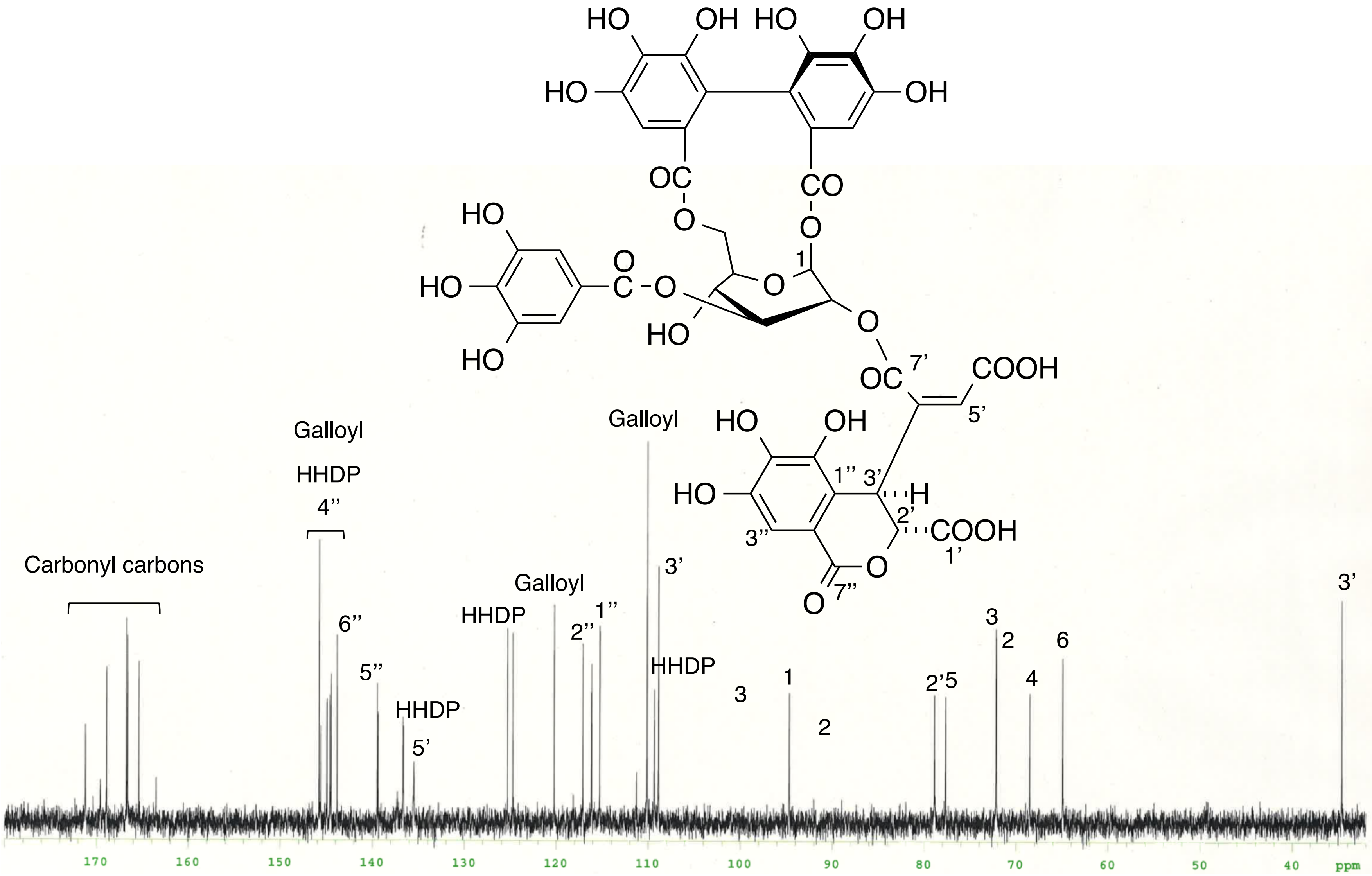

Figure S2. ^{13}C NMR spectrum of davicratinic acid A (**5**) in acetone-d_6/D_2O=9/1.

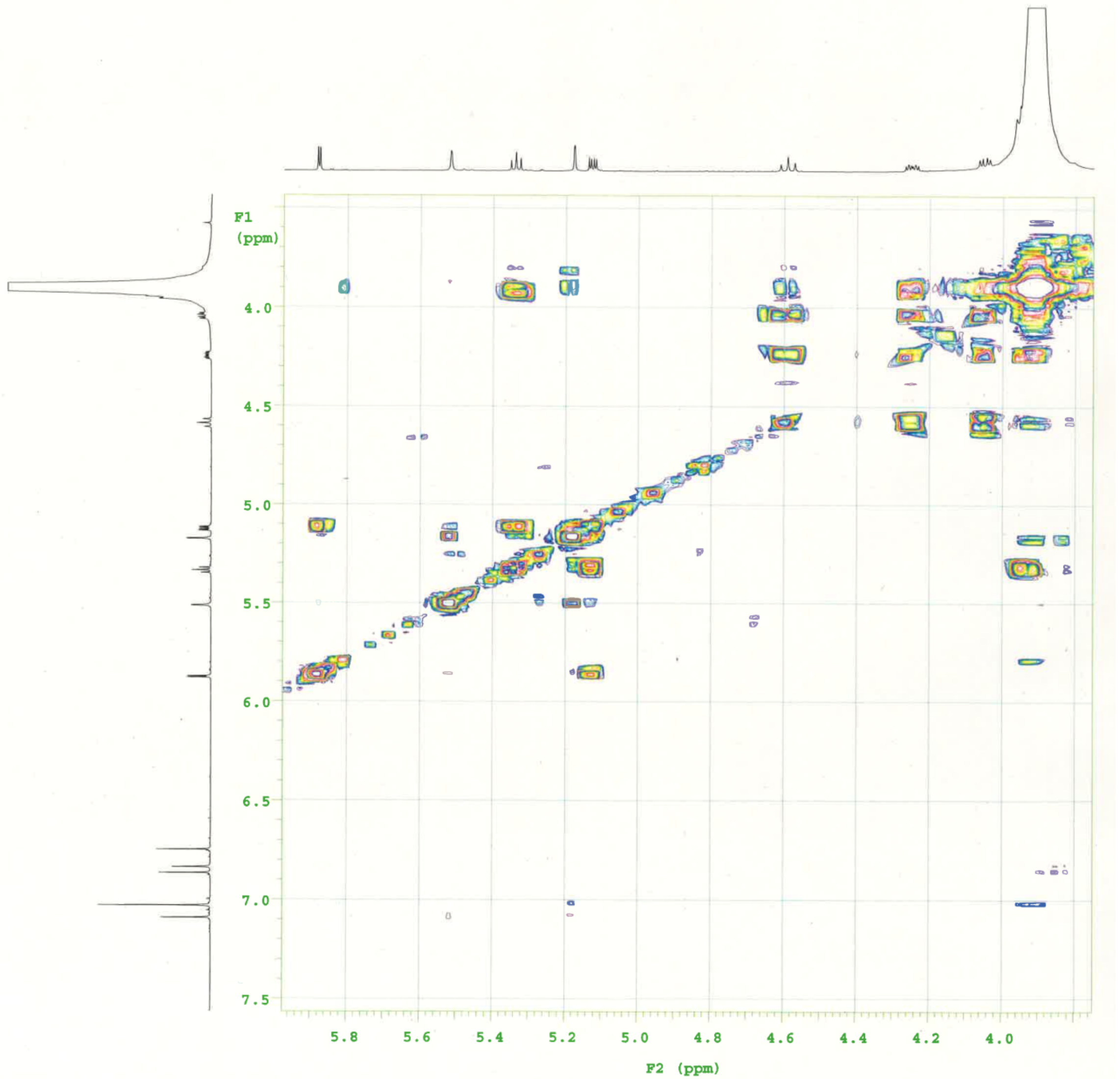

Figure S3. ^{1}H-^{1}H COSY spectrum of davicratinic acid A (**5**) in acetone-d_6/D_2O=9/1.

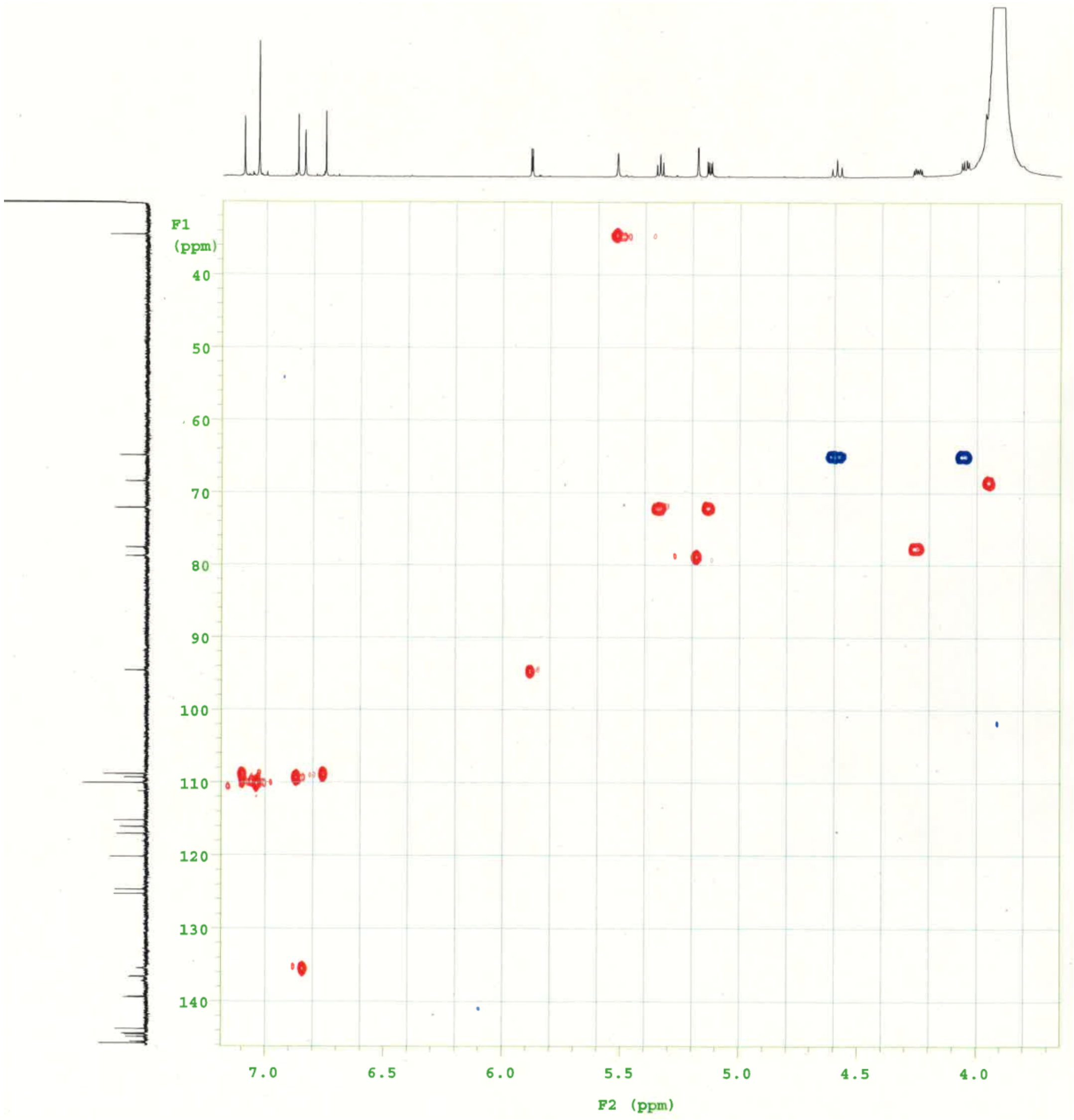

Figure S4. HSQC spectrum of davicratinic acid A (**5**) in acetone-d_6/D_2O=9/1.

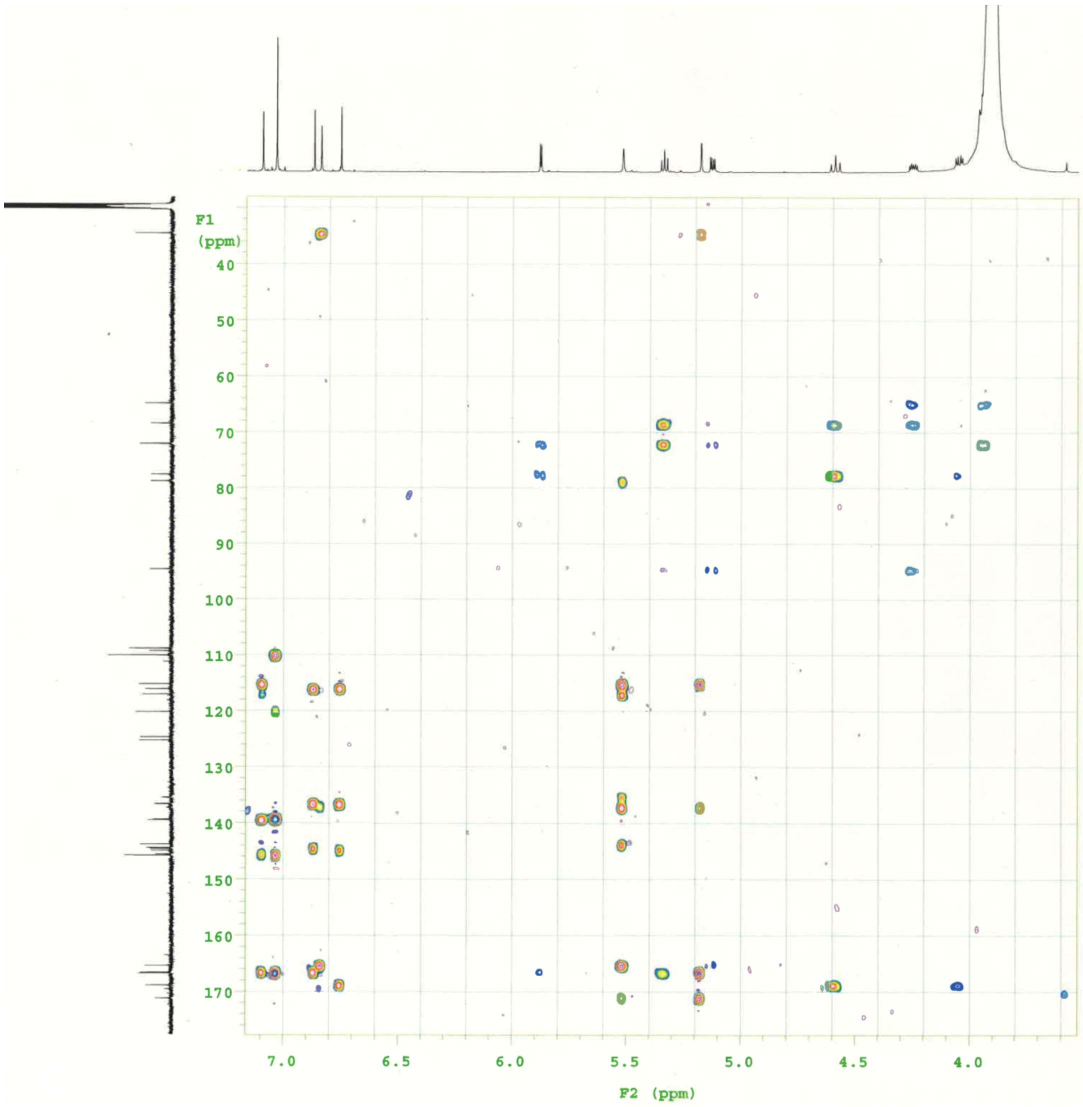

Figure S5. HMBC spectrum of davicratinic acid A (**5**) in acetone-d_6/D_2O=9/1.

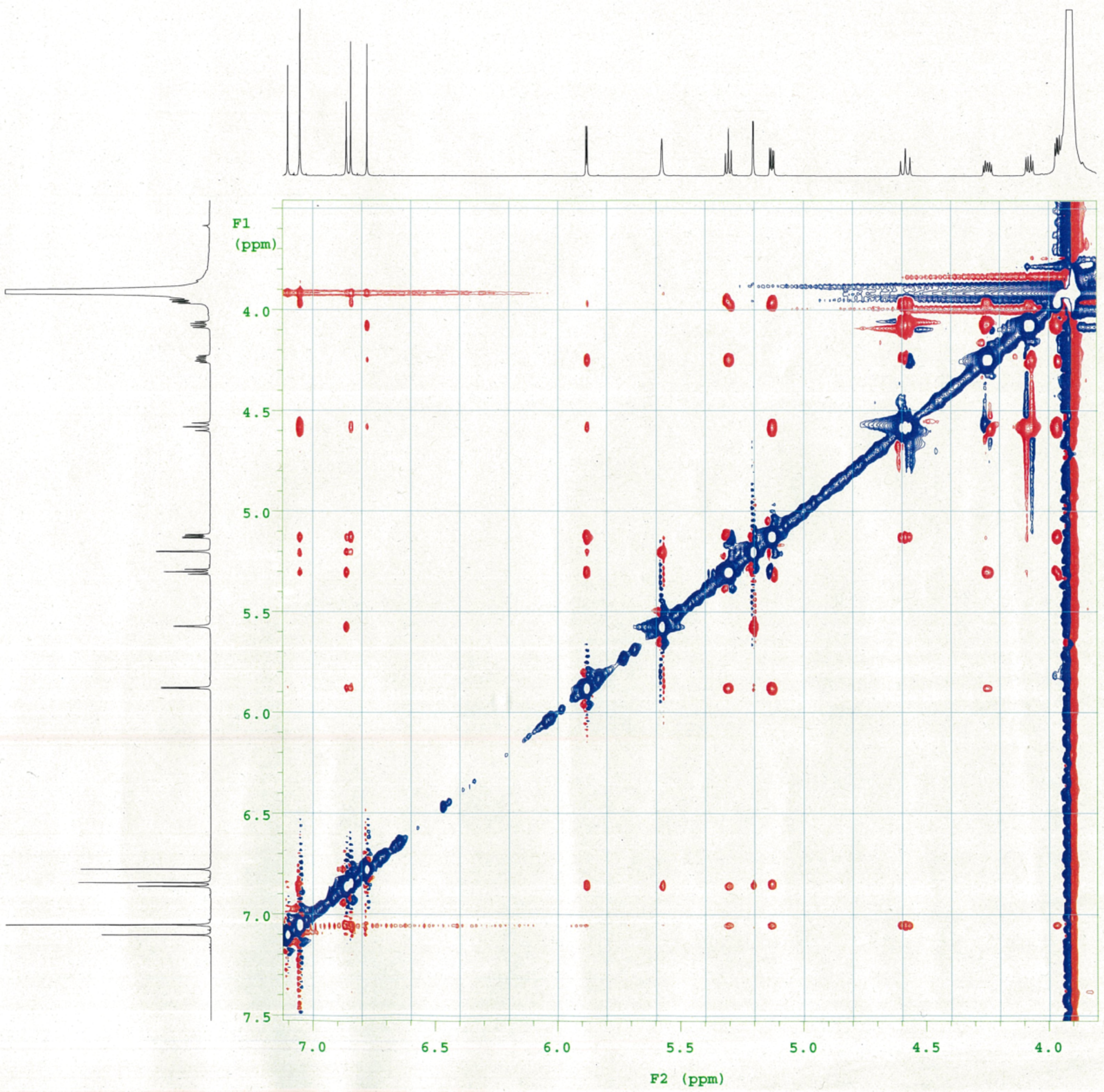

Figure S6. ROESYspectrum of davicratinic acid A (**5**) in acetone-d_6/D_2O=9/1.

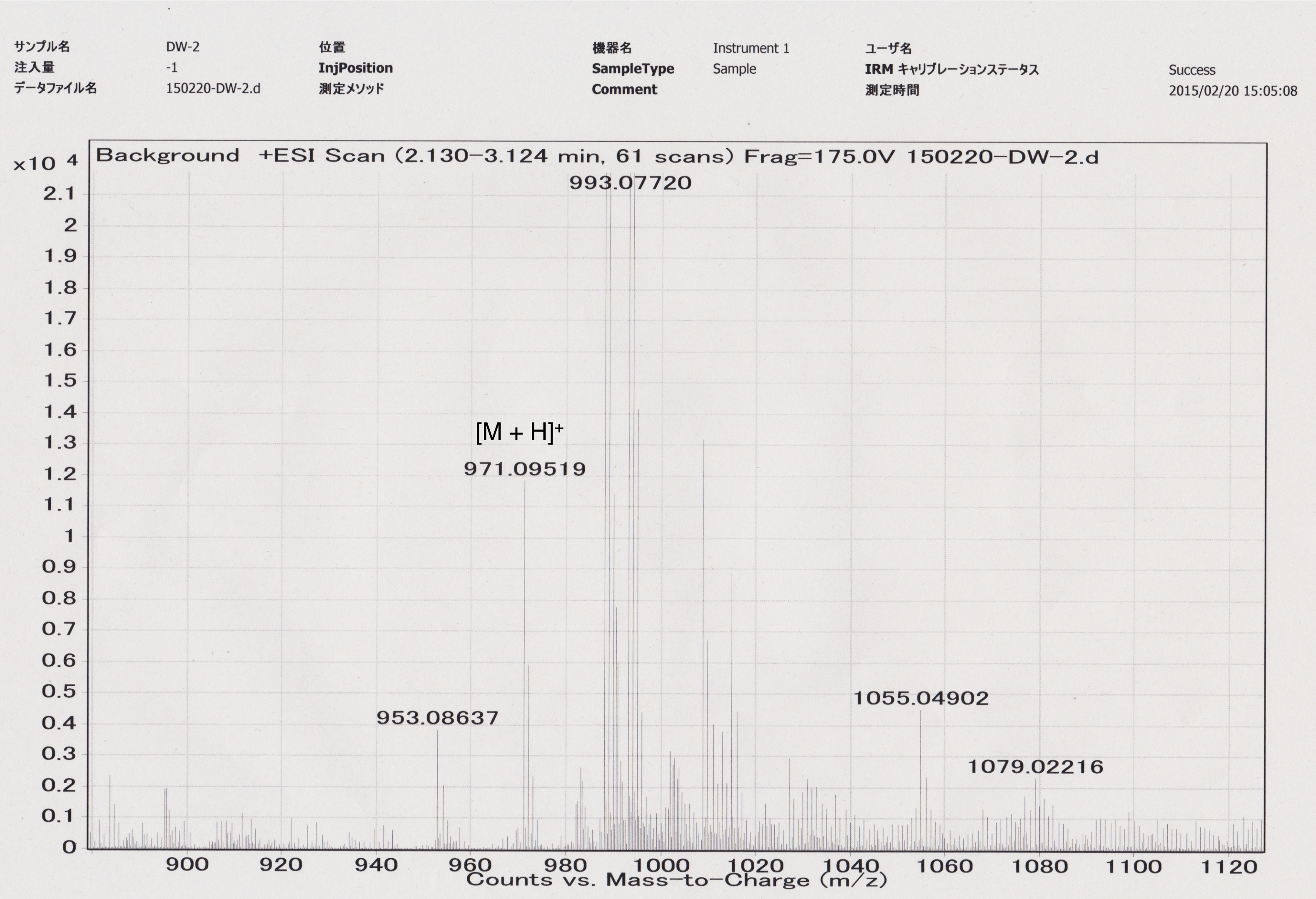

Figure S7. (+)-HR-ESI-MS spectrum of davicratinic acid A (**5**).

Table S1. Cytotoxicity of *Davidia* tannins.

1 Ca9-22

µM	Viable cell number (% of control)			mean
25				
50	99	89	93	
100	77	69	64	
200	18	23	17	
400	21	15	15	
CC_{50}	146	144	130	140

4 Ca9-22

µM	Viable cell number (% of control)			mean
25	87	91	78	
50	82	75	69	
100	53	49	44	
200	6	6	4	
400				
CC_{50}	106	98	88	97.3

Resveratrol Ca9-22

µM	Viable cell number (% of control)			mean
25				
50	64	65	63	
100	54	50	52	
200	21	17	19	
400				
CC_{50}	112	100	106	106

1 HSC-2

µM	Viable cell number (% of control)			mean
25				
50	86	83	78	
100	57	54	50	
200	25	27	24	
400	9	8	7	
CC_{50}	122	115	100	112.3

4 HSC-2

µM	Viable cell number (% of control)			mean
25				
50	84	77	80	
100	57	54	54	
200	10	11	11	
400				
CC_{50}	115	109	109	111

Resveratrol HSC-2

µM	Viable cell number (% of control)			mean
25	92	91	90	
50	69	69	70	
100	23	23	23	
200	6	5	5	
400				
CC_{50}	71	70.6	71.3	71

1 HSC-3

µM	Viable cell number (% of control)			mean
25	105	93	88	
50	79	59	72	
100	52	48	50	
200	28	29	28	
400	9	11	12	
CC_{50}	108	90.9	100	99.6

4 HSC-3

µM	Viable cell number (% of control)			mean
25				
50	82	80	82	
100	62	50	56	
200	20	18	17	
400	7	11	10	
CC_{50}	129	100	115	114.7

Resveratrol HSC-3

µM	Viable cell number (% of control)			mean
25	90	89	89	
50	65	65	67	
100	19	25	23	
200	3	4	5	
400				
CC_{50}	66.3	68.8	67.9	67.7

1 HSC-4

µM	Viable cell number (% of control)			mean
25				
50	84	89	85	
100	75	68	69	
200	38	25	29	
400				
CC_{50}	168	142	148	152.7

4 HSC-4

µM	Viable cell number (% of control)			mean
25	92	83	77	
50	81	65	68	
100	60	43	39	
200	13	8	6	
400				
CC_{50}	121	84	81	95.3

Resveratrol HSC-4

µM	Viable cell number (% of control)			mean
25	66	73	72	
50	36	51	49	
100	7	11	4	
200				
400				
CC_{50}	38.3	51.3	48.9	46.2

1 HGF

µM	Viable cell number (% of control)			mean
25				
50				
100	97	81	84	
200	70	54	62	
400	9	32	23	
CC_{50}	266	236	262	254.5

4 HGF

µM	Viable cell number (% of control)			mean
25				
50				
100	80	78	81	
200	72	66	67	
400	5	5	4	
CC_{50}	133	126	127	128.7

Resveratrol HGF

µM	Viable cell number (% of control)			mean
25				
50	66	62	65	
100	66	60	59	
200	61	58	48	
400	0	0		
CC_{50}	236	228	181	215

1 HPLF

µM	Viable cell number (% of control)			mean
25				
50				
100	88	87	102	
200	75	75	79	
400	24	28	32	
CC_{50}	298	306	323	309

4 HPLF

µM	Viable cell number (% of control)			mean
25				
50	101	100	93	
100	78	86	80	
200	12	29	30	
400				
CC_{50}	142	163	160	155

Resveratrol HPLF

µM	Viable cell number (% of control)			mean
25				
50	83	84	83	
100	74	70	71	
200	56	47	46	
400	5	1	5	
CC_{50}	224	187	184	198.3

1 HPC

µM	Viable cell number (% of control)			mean
25				
50				
100	81	87	88	
200	54	62	59	
400	20	32	31	
CC_{50}	224	280	264	256

4 HPC

µM	Viable cell number (% of control)			mean
25				
50	88	87	86	
100	67	72	71	
200	11	6	6	
400				
CC_{50}	130	133	130	131

Resveratrol HPC

µM	Viable cell number (% of control)			mean
25				
50				
100	79	76	79	
200	50	55	55	
400	7	7	8	
CC_{50}	200	221	221	214
